# Staged Orthoplastic Soft-Tissue Reconstruction of Complex Tibial Fracture-Related Infection Using a Chimeric Anterolateral Thigh Free Flap After Failed Soleus Muscle Flap: A Case Report

**DOI:** 10.3390/jcm15207804

**Published:** 2026-10-10

**Authors:** Chang-Sheng Liao, Xia Chen, Feng-Jun Lan, Huan-Sheng Liu, Qiang Huang, Yong-Xi Fan

**Affiliations:** Department of Orthopaedics, West China Hospital, Sichuan University, Chengdu 610041, China; 15603441409@163.com (C.-S.L.); 13438502627@163.com (X.C.); 17340206341@163.com (F.-J.L.); lhs200006@163.com (H.-S.L.)

**Keywords:** fracture-related infection, tibia, anterolateral thigh flap, dead-space management, case report

## Abstract

A complex tibial fracture-related infection (FRI) after local-flap failure may require staged source control, dead-space management, and vascularized soft-tissue reconstruction. A 60-year-old man with type 2 diabetes developed FRI after an industrial injury causing a localized incomplete open tibial fracture with preserved alignment and no large segmental bone defect. Previous debridements, cement-based dead-space management, negative-pressure wound therapy, and soleus muscle-flap transfer failed. On 4 June 2026, repeat radical debridement was followed by temporary vancomycin-loaded polymethylmethacrylate for dead-space management. On 25 June, the cement was removed, repeat debridement was performed, and a 16 cm × 7 cm defect with exposed tibia and tendon and a large proximal dead space was reconstructed using a chimeric anterolateral thigh–vastus lateralis free flap. No additional skeletal reconstruction was required. At 9.5-week follow-up, the flap remained fully viable with stable coverage; CRP was 2.0 mg/L, IL-6 was 3.8 pg/mL, and ESR was 22 mm/h. This case illustrates a staged orthoplastic salvage strategy after local-flap failure. Longer follow-up is required to confirm definitive infection control, fracture union, and function.

## 1. Introduction

A fracture-related infection (FRI) is a serious complication of musculoskeletal trauma and is particularly challenging after open tibial injury because contamination, exposed bone, compromised local perfusion, and limited soft-tissue coverage may coexist. International consensus criteria distinguish confirmatory findings, including wound breakdown or sinus communication, purulent drainage, repeated recovery of phenotypically indistinguishable organisms from separate deep specimens, and histopathological evidence of microorganisms, from suggestive but nonspecific findings such as elevated serum inflammatory markers [1,2]. The burden of infection after tibial fracture is substantial. A 2024 systematic review and meta-analysis of 42 studies including 24,610 surgically managed tibial fractures identified 2418 surgical-site infections (9.8%); open fracture, Gustilo–Anderson grade >2, compartment syndrome, smoking, higher ASA class, external fixation, blood transfusion, polytrauma, and high-energy injury were among the reported risk factors [3]. A separate meta-analysis of open tibial fractures estimated a deep-infection incidence of approximately 12.2 infections per 100 person-years [4], while a recent review specifically highlights the high FRI burden associated with open tibial fractures [5].

Successful FRI management requires coordinated source control, representative deep sampling, appropriate antimicrobial therapy, assessment of fracture stability and bone loss, dead-space management, host optimization, and restoration of a durable vascularized soft-tissue envelope [6,7,8,9]. In the tibia, repeated operations may leave exposed bone or tendon together with an irregular three-dimensional cavity. When local tissue options are depleted or have failed, free tissue transfer can import well-vascularized tissue from outside the zone of injury. A chimeric ALT construct can combine a skin paddle with a separately mobile vascularized vastus lateralis muscle component on the same source vascular system, allowing broad surface resurfacing and deep dead-space obliteration to be addressed independently [10,11,12].

We report a complex tibial FRI after an industrial open injury, repeated debridements, antibiotic-cement treatment, prolonged wound management, and failure of a soleus muscle flap. The chimeric ALT–vastus lateralis configuration itself is not presented as a novel technique. Rather, the case illustrates the clinical decision sequence of staged source control, temporary local antibiotic dead-space management, interpretation of changing microbiological findings, host optimization, CTA-assisted vascular planning, and definitive vascularized soft-tissue coverage in a limb with an incomplete but radiographically aligned tibial fracture that did not require additional skeletal reconstruction. The report was prepared in accordance with the CARE guidelines [13], and the completed CARE checklist is provided as Supplementary Materials.

## 2. Case Presentation

### 2.1. Initial Injury and Previous Treatment

A 60-year-old man with type 2 diabetes mellitus and hypertriglyceridemia sustained a work-related lacerating injury to the right lower leg while using an angle grinder to cut a drainage pipe in approximately mid-March 2026. He experienced immediate pain, swelling, and active bleeding. At the initial emergency visit, the wound was treated with simple debridement and primary closure. During subsequent dressing changes, progressive skin and soft-tissue necrosis developed, with tibial exposure and persistent drainage.

Approximately 19 days after the injury, he returned to the local hospital and was diagnosed with a localized incomplete open tibial fracture. This was not a complete displaced tibial shaft fracture. Subsequent review of the available radiographs and CT confirmed preservation of overall tibial alignment and longitudinal continuity, without major displacement or a large segmental osseous defect. On 3 April 2026, he underwent wound debridement, cement filling/coverage of the local wound dead space, and vacuum-assisted closure. Repeat debridement, cement-spacer placement, and NPWT were performed on 10 April. On 17 April, another debridement and soleus muscle-flap transfer were undertaken; the transferred soleus flap subsequently became necrotic and infected. He was then admitted to a tertiary center on 11 May for persistent tibial and deep soft-tissue infection, where further debridement of infected bone and soft tissue with antibiotic-cement coverage was performed. Meropenem plus vancomycin was documented during this phase. Serial cultures at that center yielded *Corynebacterium tuberculostearicum* on 12 May, *Bacteroides fragilis* on 13 May, and *B. fragilis* together with *Staphylococcus epidermidis* on 16 May. C-reactive protein (CRP) was 16.8 mg/L on 12 May, 15.6 mg/L on 16 May, and increased to 64.1 mg/L on 18 May; glycated hemoglobin was 8.0% on 14 May.

### 2.2. Orthopedic Assessment After Transfer and Preoperative Vascular Planning

The patient was transferred to the treating orthoplastic unit on 20 May 2026 for persistent right lower-leg FRI and a residual soft-tissue defect after multiple previous operations. Examination showed an approximately 8 cm × 6 cm defect over the medial middle-to-distal lower leg, with antibiotic cement occupying the wound bed. The surrounding tissues were markedly edematous and erythematous, with a small amount of bloody exudate. A proximal approximately 15 cm surgical incision was present. The ankle and foot were substantially swollen. Dorsalis pedis pulsation was palpable, posterior tibial pulsation was diminished, and distal toe perfusion remained clinically acceptable. Knee and ankle active motion was limited by pain.

Early laboratory testing after transfer showed CRP was 21.9 mg/L (reference range, 0–6.0 mg/L), erythrocyte sedimentation rate (ESR) was 120 mm/h (reference range, <21 mm/h), hemoglobin was 69 g/L (reference range, 130–175 g/L), and serum albumin was 35.8 g/L (reference range, 40.0–55.0 g/L). Diabetes, severe anemia, nutritional status, thrombosis risk, and limb perfusion were addressed during preoperative optimization. Review of the available radiographs and CT demonstrated a localized incomplete cortical fracture/defect of the mid-to-distal tibial diaphysis adjacent to the injured soft-tissue envelope. Overall tibial alignment and longitudinal continuity were preserved, without major displacement, clinically relevant angulation or shortening, or a large segmental osseous defect. No internal or external fixation was present. Given the limited, incomplete fracture morphology, preserved alignment and continuity, and absence of a large segmental bone defect, no fracture fixation, bone grafting, bone transport, or other additional skeletal reconstruction was considered necessary during the reported treatment period. The principal orthopedic-reconstructive problems were therefore infection source control, exposed tibia, devitalized soft tissue, and dead-space management rather than correction of a major fracture deformity.

**Figure 1:**
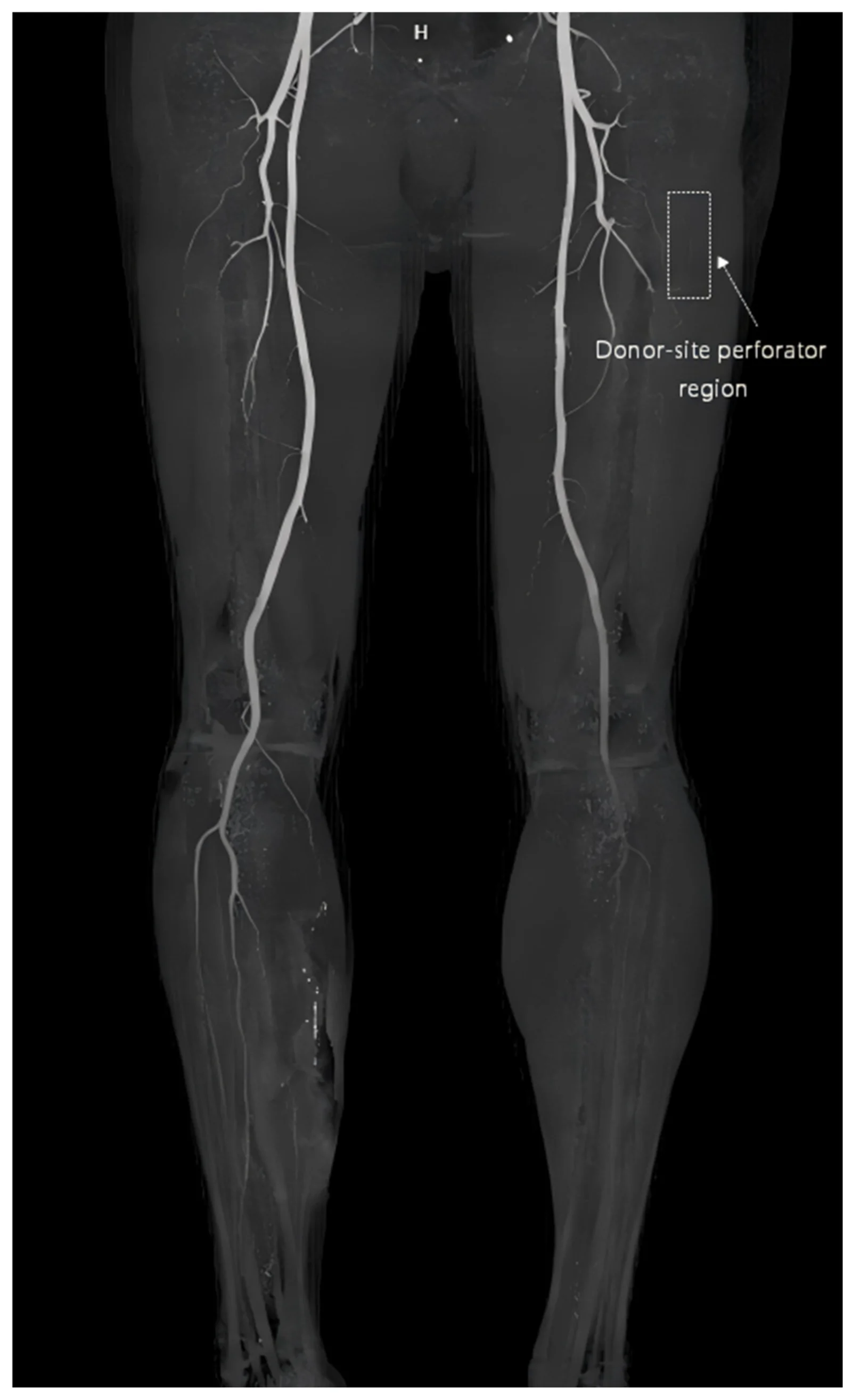
Preoperative computed tomography angiography (CTA) for donor-site vascular assessment. Coronal lower-extremity CTA demonstrates the overall arterial anatomy and the region of the planned donor-site perforator in the left thigh. The letter H is a workstation-generated orientation marker indicating the cranial (head) direction.

**Figure 2:**
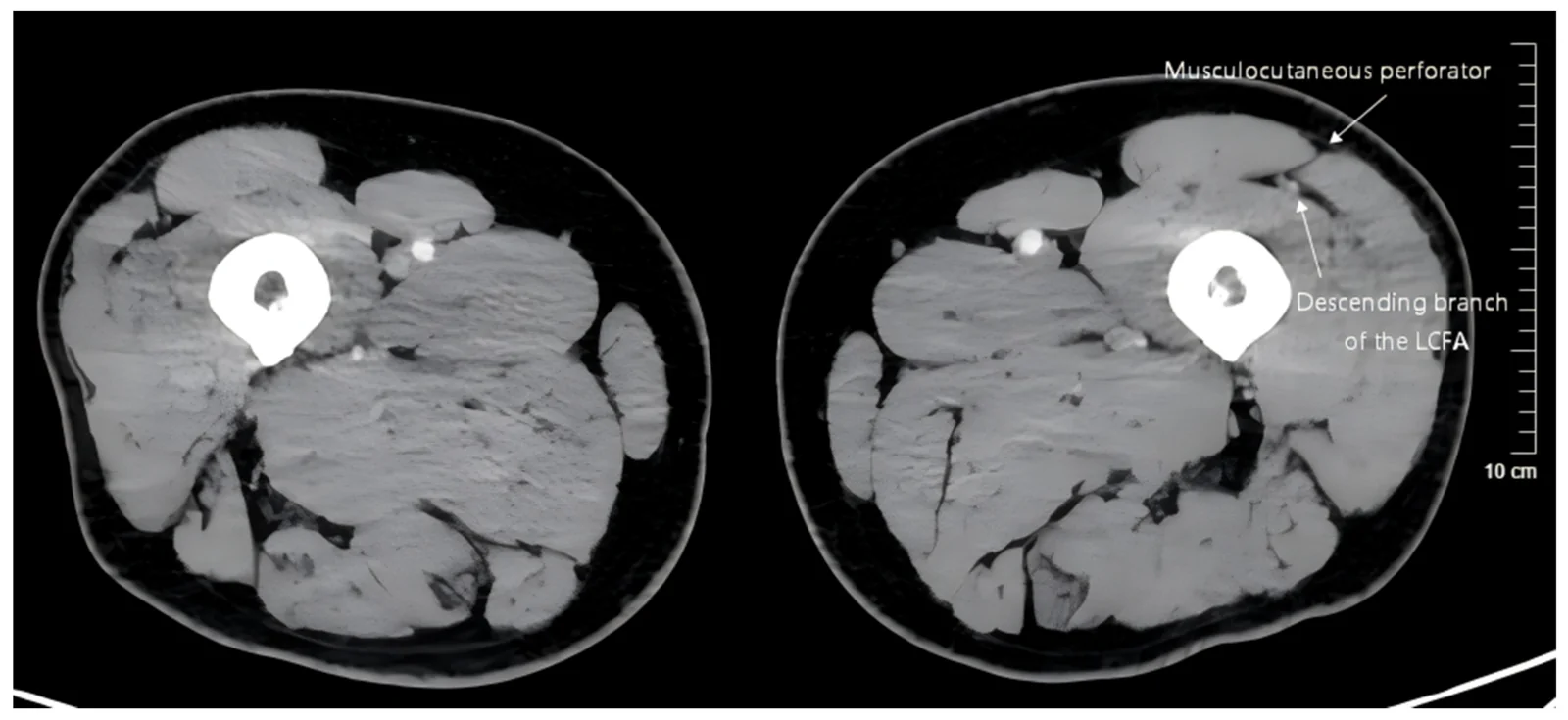
Axial CTA assessment of the left thigh donor site. The annotated image demonstrates a musculocutaneous perforator and its relationship to the descending branch of the lateral circumflex femoral artery (LCFA), supporting preoperative planning of the chimeric ALT flap. The central high-attenuation white areas represent the femoral cortical bone on axial CTA.

Because the patient had undergone repeated debridements and a failed local muscle flap and was being considered for free tissue transfer, lower-extremity CTA with vascular reconstruction was obtained to assess recipient- and donor-site vascular anatomy. Coronal CTA demonstrated the overall lower-extremity arterial course and the donor-site perforator region (Figure 1). Axial CTA of the thigh further demonstrated the relationship between the descending branch of the lateral circumflex femoral artery (LCFA) and a musculocutaneous perforator coursing toward the superficial soft tissues (Figure 2). These findings were integrated with wound geometry and intraoperative recipient-vessel exploration rather than used as a stand-alone determinant of flap selection [14].

### 2.3. Sequential Microbiology and Antimicrobial Management

The microbiological profile changed during the staged treatment course. At the tertiary center before transfer, serial cultures yielded *Corynebacterium tuberculostearicum* on 12 May, *Bacteroides fragilis* on 13 May, and *B. fragilis* together with *Staphylococcus epidermidis* on 16 May. The repeated recovery of *B. fragilis* was considered clinically plausible in the context of a contaminated traumatic wound and persistent deep infection. Following repeat treatment, subsequent operative/microbiology reports included *Bacillus cereus*, *Bacillus subtilis*, *Paenibacillus* spp., *Staphylococcus haemolyticus*, and *Staphylococcus warneri*. *Corynebacterium* species, coagulase-negative staphylococci, and environmental spore-forming organisms were interpreted according to specimen source, repeated recovery, operative findings, and clinical concordance rather than automatically being labeled as separate new infections [2,15]. The working diagnosis remained clinically definite FRI with a dynamic polymicrobial culture profile during repeated debridement and antimicrobial exposure.

Systemic antimicrobial therapy was adjusted according to prior cultures, available susceptibility data, operative findings, clinical response, and host tolerance. Before transfer, meropenem plus vancomycin was documented, although exact doses from that phase were not available in the transferred records. After transfer, medication orders documented vancomycin 0.5 g intravenously every 12 h from 20 to 26 May, norvancomycin 0.8 g intravenously every 12 h from 26 to 28 May, and levofloxacin 0.5 g intravenously once daily from 27 May to 1 June. Levofloxacin 0.5 g intravenously once daily was restarted from 22 June to 13 July, and linezolid 600 mg intravenously every 12 h was administered from 23 to 30 June during the peri-reconstruction period. The patient was already receiving therapeutic antimicrobial treatment during the principal staged procedures; no separate perioperative prophylactic regimen was clearly documented, and none was inferred. Local vancomycin-loaded PMMA was used on 4 June as a temporary adjunct for dead-space management. The treatment strategy emphasized deep sampling at decisive surgical stages and avoided indefinite escalation solely to cover every organism previously reported (Table 1).

### 2.4. Stage 1: Repeat Source Control and Dead-Space Management

Because the previous soleus flap had failed and residual dead space, devitalized tissue, and infection risk persisted, definitive vascularized soft-tissue coverage was not performed immediately. On 4 June 2026, the patient underwent repeat debridement of chronically infected tibial and soft tissues, excision of nonviable tissue, antibiotic-cement dead-space management, and NPWT. Intraoperatively, an approximately 6 cm × 6 cm medial mid-leg defect was present. Proximally, a post-soleus-flap cavity measuring approximately 20 cm × 5 cm contained cement, blood clot, scattered devitalized muscle, and inflammatory granulation tissue.

**Figure 3:**
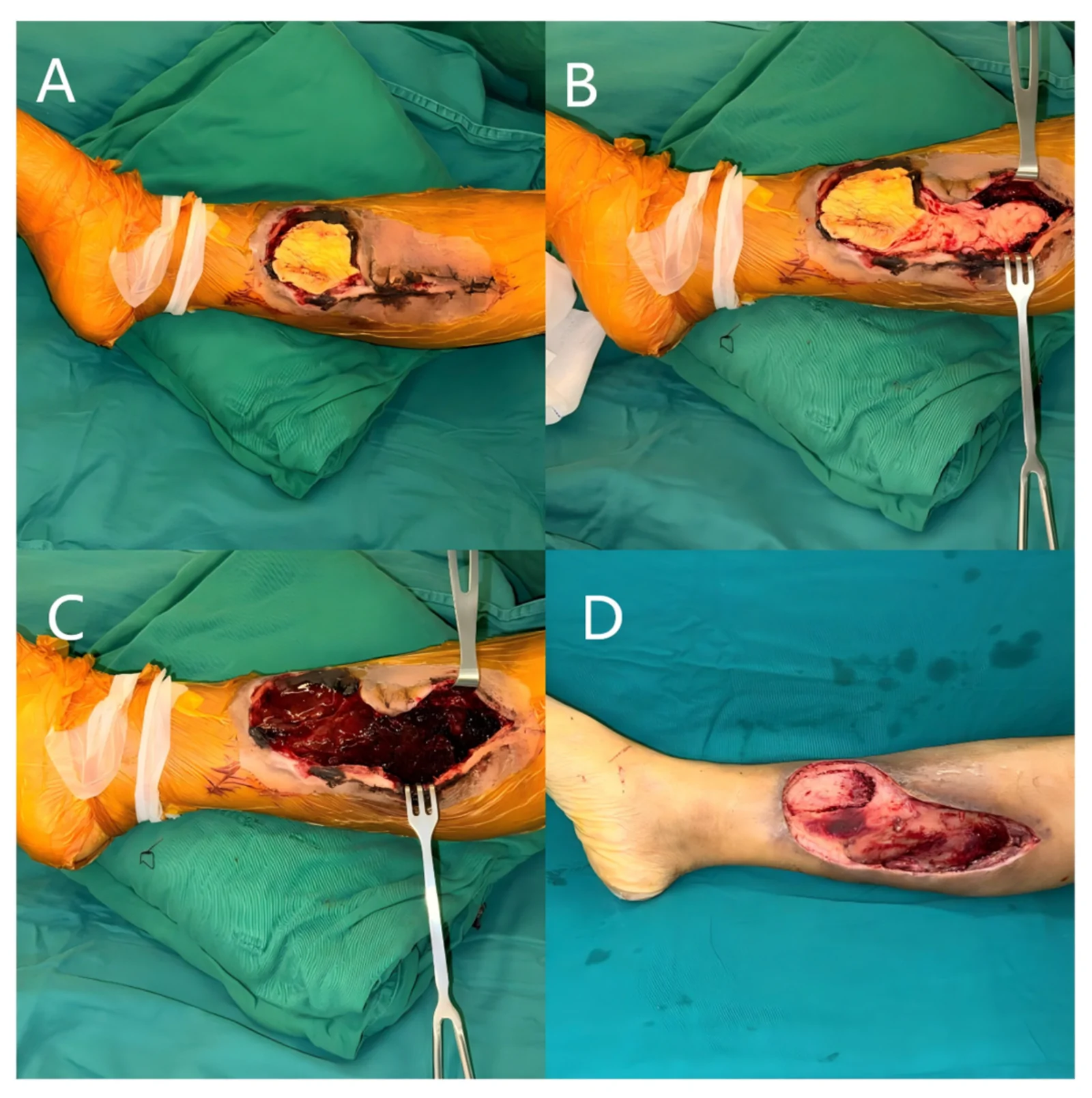
Staged source control and wound preparation. (**A**) Medial right lower-leg wound before repeat debridement, with antibiotic cement and compromised surrounding soft tissues; (**B**) wound after removal of the existing cement, exposing the deep defect and residual cavity; (**C**) wound after further debridement of devitalized tissue; (**D**) recipient defect before definitive free-flap reconstruction, demonstrating extensive soft-tissue loss and exposed deep structures.

The existing antibiotic cement was removed. Blood clot, suspicious infected tissue, necrotic tissue, and inflammatory granulation tissue were excised, and deep tissue was submitted for microbiological culture. After repeated irrigation and re-preparation, vancomycin-loaded polymethylmethacrylate (PMMA) was used to occupy the residual cavity and cover the exposed tibial region. VAC was then applied as temporary wound management. The PMMA placed at this stage was explicitly intended as temporary local antimicrobial and dead-space management during the interval before definitive vascularized soft-tissue coverage, rather than as definitive skeletal reconstruction (Figure 3).

### 2.5. Interval Optimization and Reconstructive Decision

During the interval between staged procedures, anemia, nutritional status, glycemic control, inflammatory activity, and wound condition were reassessed. By 18 June, hemoglobin had increased to approximately 115 g/L, CRP was approximately 6.6 mg/L, and interleukin-6 was 4.54 pg/mL; on 22 June, CRP was approximately 7.4 mg/L and interleukin-6 was 2.21 pg/mL. The decision to proceed to vascularized soft-tissue coverage was based on repeated source control, removal of grossly devitalized tissue, a clinically viable wound bed, improved host status, preserved skeletal alignment, and reconstructible vascular anatomy. Culture negativity was not used as an isolated prerequisite.

### 2.6. Stage 2: Definitive Vascularized Soft-Tissue Coverage with a Chimeric ALT Free Flap

On 25 June 2026, the temporary vancomycin-loaded PMMA placed on 4 June was removed. Repeat wound assessment and debridement were then performed before vascularized soft-tissue coverage. The right medial proximal-to-mid lower leg had an approximately 16 cm × 7 cm soft-tissue defect with exposed tibia and adjacent tendon, together with a substantial proximal dead space. Inflammatory granulation tissue and partially devitalized muscle were further excised, and no obvious gross purulent secretion was recorded at the time of reconstruction.

**Figure 4:**
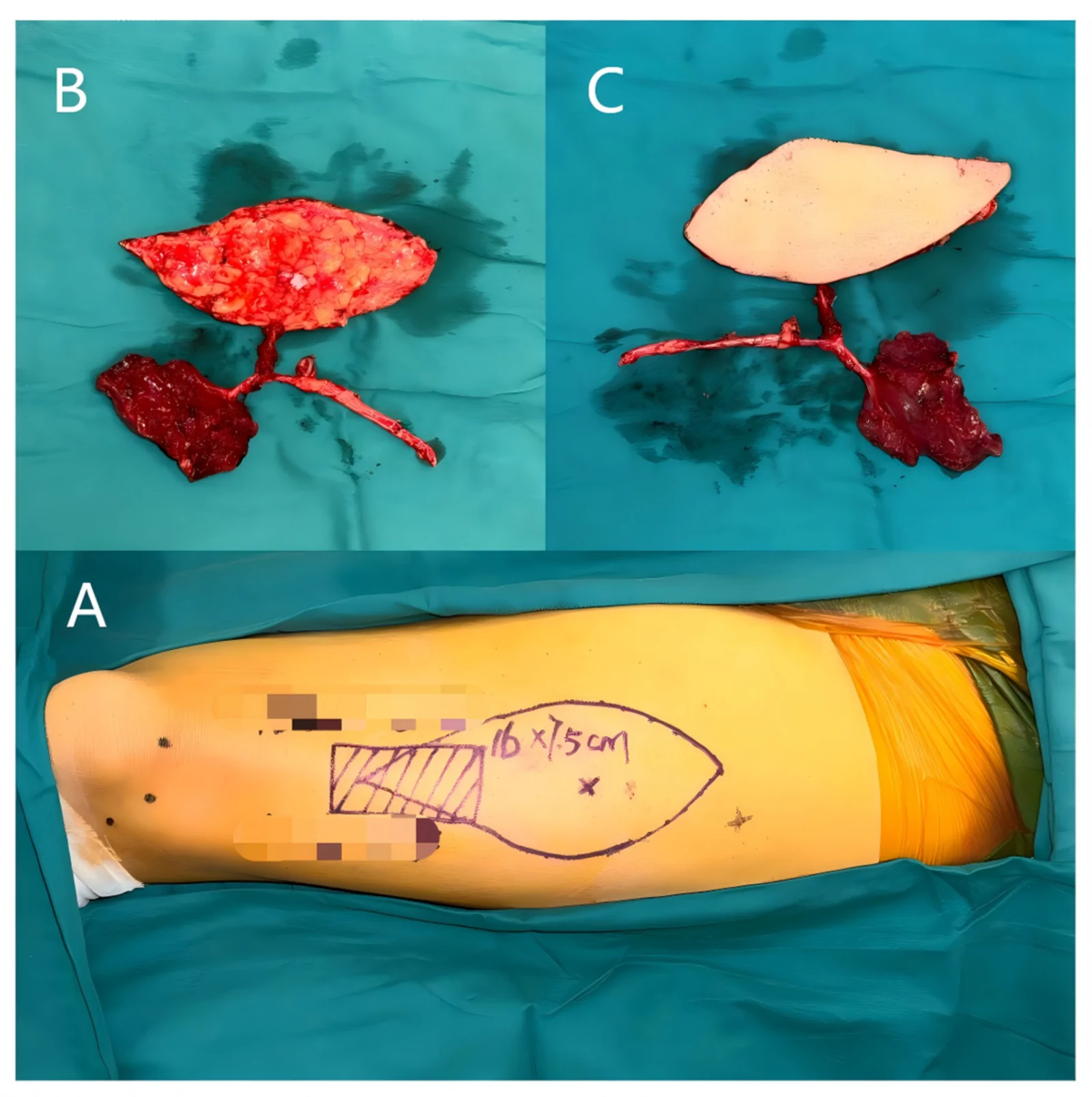
Design and harvest of the chimeric ALT free flap. (**A**) Left anterolateral thigh donor-site design centered on the selected perforator; an identifying handwritten number was digitally obscured for privacy. (**B**) Deep surface of the harvested chimeric flap, demonstrating the skin/subcutaneous component, a separately mobile muscle component, and the common vascular system. (**C**) Cutaneous surface and pedicle relationship of the chimeric construct. The configuration allowed the muscle component to fill the deep cavity while the skin paddle provided surface coverage.

A template of the recipient defect was transferred to the contralateral anterolateral thigh. A perforator-centered ALT skin paddle was designed around the CTA-identified donor region, and the perforator was dissected retrogradely toward the descending branch of the LCFA. Because the recipient wound had both a broad surface defect and a deep three-dimensional cavity, a separate segment of vascularized vastus lateralis muscle was harvested on a muscular branch arising from the same source vascular system, producing a chimeric free tissue flap. The skin and muscle components could therefore be positioned independently while sharing the source pedicle (Figure 4).

The composite flap was transferred to the right lower-leg defect. The vascularized muscle component was inset into the proximal dead space, whereas the skin paddle restored external coverage of the exposed tibia, tendon, and soft-tissue defect. The anterior tibial artery and two venae comitantes were selected as recipient vessels. Microsurgical arterial and venous anastomoses were completed, after which arterial inflow and venous outflow were satisfactory. The lateral femoral cutaneous nerve carried with the flap was coapted end-to-end to a recipient cutaneous sensory nerve with the intention of sensory restoration. At completion of surgery, flap color, tension, capillary bleeding, and needle-prick response were satisfactory. A donor-site drain was placed, limited NPWT was used around residual wound areas as appropriate, and the ankle was immobilized in a neutral-position cast.

### 2.7. Early Postoperative Course and Short-Term Clinical and Radiographic Follow-Up

**Figure 5:**
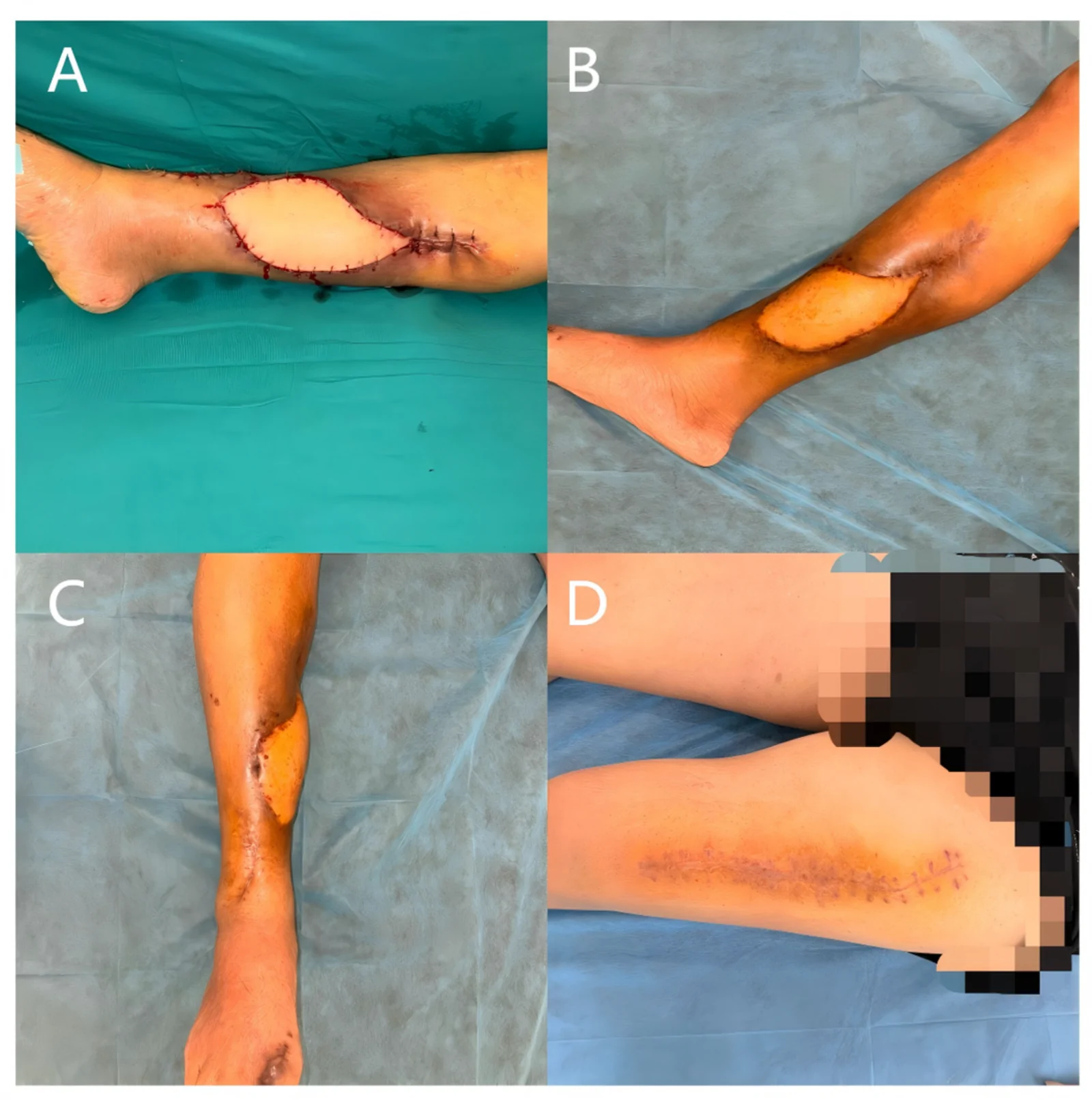
Recipient- and donor-site appearance after chimeric ALT reconstruction. (**A**) Early postoperative appearance after flap inset. (**B**,**C**) Recipient-site appearance at outpatient follow-up on 10 August 2026, approximately 6.5 weeks after reconstruction, showing a viable flap and stable soft-tissue coverage. (**D**) Left anterolateral thigh donor-site appearance at the same follow-up visit.

The early postoperative course demonstrated maintained flap viability and stable recipient-site coverage. On 3 July 2026, ESR had decreased to approximately 15 mm/h and CRP to approximately 7.3 mg/L. A transient inflammatory rebound was documented on 11 July, with ESR 120 mm/h and CRP 44.3 mg/L. The patient remained clinically stable with a viable flap and no documented recurrent wound breakdown or drainage (Figure 5). After clinical stabilization, he was discharged from our center on 13 July and transferred to a lower-level hospital for continued systemic antimicrobial therapy. At outpatient follow-up on 10 August 2026, approximately 6.5 weeks after reconstruction, the flap remained viable with stable soft-tissue coverage and no recurrent wound breakdown or drainage. CRP was 6.9 mg/L, ESR was 44 mm/h, hemoglobin was 112 g/L, white blood cell count was 4.27 × 10^9^/L, and serum albumin was 43.7 g/L.

**Figure 6:**
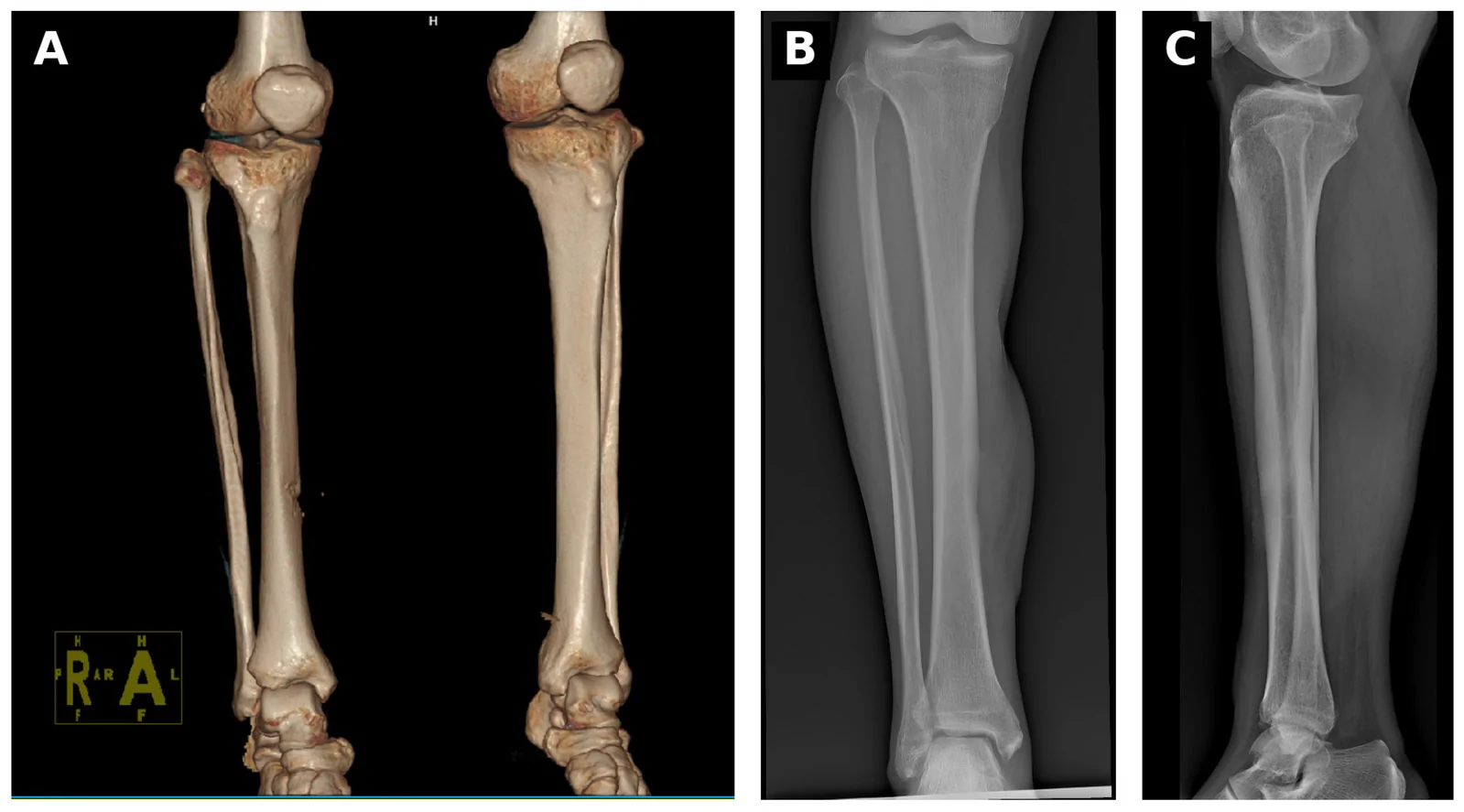
Orthopedic imaging of the right tibia. (**A**) Three-dimensional CT reconstruction obtained during tertiary-center treatment demonstrating a localized incomplete cortical tibial defect with preserved overall alignment and longitudinal continuity and without a large segmental osseous defect. (**B**,**C**) Anteroposterior and lateral radiographs obtained at outpatient follow-up on 10 August 2026 show preserved overall tibial alignment and longitudinal continuity, with a stable radiographic appearance of the localized incomplete fracture. Because follow-up was short, definitive radiographic union is not claimed.

Follow-up anteroposterior and lateral radiographs obtained on 10 August demonstrated preserved overall tibial alignment and longitudinal continuity, with a stable radiographic appearance of the localized incomplete fracture (Figure 6B,C). No internal or external fixation had been required. Because of the short follow-up interval, definitive fracture union was not claimed.

At the subsequent outpatient follow-up on 30 August 2026, approximately 9.5 weeks after free-flap reconstruction, the chimeric ALT flap remained fully viable, and the reconstructed soft-tissue envelope remained stable. Laboratory testing showed CRP was 2.0 mg/L and IL-6 was 3.8 pg/mL, both within the institutional reference ranges, while ESR had decreased to 22 mm/h, close to the reference limit. These findings indicated marked overall improvement in the inflammatory profile and supported sustained short-term reconstructive success. Nevertheless, the available follow-up remains insufficient to establish definitive FRI eradication, complete fracture union, durable sensory recovery, full weight-bearing recovery, or long-term limb salvage. Continued clinical, laboratory, and radiographic surveillance is required.

### 2.8. Summary of Clinical Course

The major clinical events, staged procedures, microbiological findings, and follow-up milestones are summarized chronologically in Table 2.

## 3. Discussion

### 3.1. Definite FRI with a Dynamic Microbiological Profile

The diagnosis of FRI in this patient was based on an open tibial fracture followed by wound breakdown, persistent drainage, exposed bone, repeated operative debridement, and deep infection findings; it did not depend on a single serum biomarker or a single culture result. CRP and ESR are suggestive rather than confirmatory criteria and may fluctuate during staged treatment [1,2]. The postoperative inflammatory rebound therefore needed to be interpreted together with local wound findings, operative assessment, microbiology, and systemic events.

The culture record was complex but clinically informative. *Bacteroides fragilis* was recovered on two separate dates before transfer, supporting its plausibility as a pathogen in a contaminated traumatic wound. By contrast, *Corynebacterium* species and coagulase-negative staphylococci are frequent skin commensals, and the clinical significance of single positive cultures depends strongly on specimen source, repeated recovery, and clinical concordance [15]. Later reports included *Bacillus* and *Paenibacillus* species and additional coagulase-negative staphylococci. Repeated surgery, NPWT, local cement, prolonged hospitalization, and systemic antimicrobial pressure may alter the local microbial ecology. The practical message is therefore not that each isolate represented a discrete new infection, but that deep sampling, specimen quality, repeated recovery, operative findings, and clinical response must be interpreted together.

### 3.2. Orthopedic Context: Incomplete Tibial Fracture Without Segmental Bone Loss

The orthopedic component of this case is important because the original injury was a localized incomplete tibial fracture rather than a complete displaced tibial shaft fracture. Review of the radiographs and CT demonstrated an incomplete cortical fracture/defect with preserved tibial alignment and longitudinal continuity, without major displacement, clinically relevant angulation or shortening, or a large segmental osseous defect. Accordingly, the clinical problem was dominated by infection, exposed tibia, devitalized soft tissue, and dead space rather than by a severe fracture deformity requiring mechanical reconstruction.

No internal or external fixation was used because the incomplete fracture remained aligned and did not show a morphology requiring additional stabilization during the reported treatment period. Likewise, bone grafting, bone transport, a Masquelet second-stage grafting procedure, or other additional skeletal reconstruction was not indicated because there was no large segmental bone defect. Short-term radiographs on 10 August showed preserved alignment and longitudinal continuity, with a stable radiographic appearance of the localized incomplete fracture. Nevertheless, the short interval precludes a claim of fracture union, and radiographic surveillance remains necessary.

### 3.3. Repeat Debridement, Temporary Local Antibiotic Cement, and Short-Term NPWT

FRI treatment principles place surgical source control at the center of management. Systemic antimicrobials cannot compensate for retained nonviable bone, devitalized soft tissue, or an unaddressed cavity [6,7,8]. The failed soleus flap in this case left a compromised local environment containing clot, necrotic muscle, inflammatory granulation tissue, and a substantial potential space. Repeat debridement on 4 June therefore preceded definitive vascularized coverage. Vancomycin-loaded PMMA provided temporary local antimicrobial delivery while occupying the dead space; importantly, it was removed on 25 June and was not intended as definitive skeletal reconstruction [8].

NPWT was used as a bridge rather than an endpoint. Current recommendations on FRI soft-tissue management emphasize avoiding prolonged reliance on NPWT and proceeding to vascularized closure when debridement is adequate, the wound bed is viable, the host is sufficiently optimized, and reconstructive conditions are suitable [9]. In this patient, the transition from temporary PMMA/NPWT to vascularized tissue was made after repeated source control and reassessment rather than after waiting for universal culture negativity.

### 3.4. CTA-Guided Planning in a Previously Operated Limb

Preoperative vascular assessment had practical value because repeated operations and inflammation could have altered the recipient environment, while the planned chimeric flap required dependable donor-side perforator anatomy. Coronal CTA provided an overview of the lower-extremity arterial tree and donor region, while axial CTA demonstrated the relationship between a musculocutaneous perforator and the descending branch of the LCFA. CTA has been used to support preoperative ALT perforator localization and flap planning [14]. In this patient, imaging was used to anticipate donor-site dissection and recipient-vessel options; final vessel selection remained an intraoperative decision.

This imaging-to-surgery sequence should be viewed as an adjunct rather than a substitute for operative judgment. CTA can reduce uncertainty regarding perforator course and donor anatomy, but vessel quality, tissue viability, pedicle reach, and anastomotic geometry must still be confirmed directly. The anterior tibial artery and two venae comitantes ultimately provided satisfactory inflow and outflow.

### 3.5. Why a Chimeric ALT Free Flap After Failed Soleus Reconstruction?

The final defect was not a planar skin defect. It combined an approximately 16 cm × 7 cm surface loss with exposed tibia and tendon and a large proximal cavity. A conventional fasciocutaneous flap could restore surface coverage but might not adequately occupy the deep dead space, whereas a muscle-only reconstruction would be less suited to broad cutaneous resurfacing and postoperative skin-paddle monitoring. The chimeric ALT configuration allowed a skin paddle and a separately mobile vastus lateralis muscle segment to be positioned independently on the same source vascular system [11]. In this patient, the muscle component filled the deep cavity while the skin component provided durable external coverage.

The choice was also influenced by failure of the previous soleus flap. Scar, inflammation, and local tissue depletion reduced the reliability and versatility of further local options. Free tissue transfer introduced well-vascularized tissue from outside the zone of injury and provided greater freedom in matching tissue components to the geometry of the defect. Clinical series have already demonstrated the versatility of chimeric ALT constructs for three-dimensional lower-extremity reconstruction and the use of ALT-based reconstruction after debridement for chronic leg osteomyelitis [11,12]. Therefore, the novelty of this report does not lie in the flap configuration itself. Its clinical value lies in showing how an established chimeric construct was incorporated into a staged salvage strategy after local-flap failure in a patient with persistent FRI, a large dead space, exposed tibia/tendon, and host compromise.

A sensory nerve was also carried with the flap and coapted to a recipient cutaneous nerve. The neural anatomy of the ALT flap permits this strategy [16]. However, performing neurorrhaphy does not itself establish meaningful sensory recovery; objective protective-sensation testing and longer follow-up are required before functional benefit can be claimed.

### 3.6. Timing of Vascularized Coverage, Clinical Implications, and Limitations

The timing of vascularized coverage deserves emphasis. Microbiology around the reconstructive period remained complex, yet delaying closure until every culture became negative would have prolonged exposure of bone and tendon. Contemporary FRI soft-tissue guidance does not support culture negativity as the sole prerequisite for closure [9]. A more coherent threshold is successful removal of grossly infected and nonviable tissue, representative deep sampling, a viable wound bed, improved host status, acceptable skeletal stability, and a reconstructive plan capable of providing vascularized coverage while systemic antimicrobial therapy is adjusted to the most credible current microbiology.

From a clinical perspective, the case supports a structured orthoplastic approach in which fracture stability, infection source control, temporary dead-space management, host optimization, microbiological reassessment, and vascularized soft-tissue coverage are considered together rather than as isolated steps. It also highlights the need to distinguish early reconstructive success from definitive infection eradication. Future prospective studies should evaluate reproducible criteria for the timing of vascularized coverage after staged debridement, clarify the clinical significance of changing low-virulence or environmental isolates during repeated treatment, and compare reconstructive strategies for complex three-dimensional tibial defects. Longer-term studies incorporating infection recurrence, radiographic fracture healing, reoperation, weight-bearing status, joint function, sensory recovery, and patient-reported outcomes are needed.

Several limitations should temper interpretation. This is a single case with relatively short follow-up after definitive vascularized soft-tissue coverage; the latest documented outpatient assessment was approximately 9.5 weeks after free-flap reconstruction. The specimen-level number and anatomical source of some later culture results were not consistently available in the extracted record, limiting organism-level causal attribution. Although a transient inflammatory rebound occurred before discharge, the patient was clinically stable with a viable flap and was transferred to a lower-level hospital for continued systemic antimicrobial therapy. By 30 August 2026, the flap remained fully viable and inflammatory indices had markedly improved (CRP 2.0 mg/L and IL-6 3.8 pg/mL within institutional reference ranges; ESR 22 mm/h, close to the reference limit). Nevertheless, this interval remains insufficient to document definitive infection eradication, complete fracture union, durable functional recovery, objective sensory recovery, or long-term limb salvage. The present report therefore supports technical feasibility and sustained short-term soft-tissue reconstructive success within a staged FRI pathway rather than completion of the entire treatment course.

## 4. Conclusions

Complex tibial FRI after repeated debridement and failure of a local soleus flap requires coordinated assessment of fracture morphology and stability, infection source, dead space, soft-tissue loss, vascular anatomy, antimicrobial treatment, and host factors. In this patient, the tibial injury was a localized incomplete fracture with preserved alignment and longitudinal continuity and without a large segmental bone defect; therefore, additional fixation, bone grafting, bone transport, or other skeletal reconstruction was not indicated. Repeat radical debridement with temporary vancomycin-loaded PMMA and short-term NPWT was followed by removal of the PMMA, repeat debridement, and definitive vascularized soft-tissue coverage using a chimeric ALT flap with a vastus lateralis muscle component. The muscle filled the deep cavity while the skin paddle restored surface coverage. The chimeric ALT technique itself is established; the principal clinical lesson is the integration of staged source control, temporary dead-space management, host optimization, vascular planning, and three-dimensional soft-tissue salvage after local-flap failure. Serial short-term follow-up demonstrated sustained flap survival, stable vascularized soft-tissue coverage, a stable radiographic appearance of the incomplete tibial fracture, and marked improvement in inflammatory indices; by 30 August 2026, CRP was 2.0 mg/L, and IL-6 was 3.8 pg/mL, both within the institutional reference ranges, while ESR was 22 mm/h, close to the reference limit. Longer follow-up is still required to determine definitive infection control, fracture union, sensory recovery, and function.

## Figures and Tables

**Table 1:** Chronology of microbiological findings and antimicrobial/surgical management.

Date/Period	Microbiology Reported	Antimicrobial/Surgical Management	Clinical Interpretation
11–18 May 2026 (tertiary center)	12 May: *Corynebacterium tuberculostearicum*; 13 May: *Bacteroides fragilis*; 16 May: *B. fragilis* + *Staphylococcus epidermidis*	Further bone/soft-tissue debridement and antibiotic-cement coverage; meropenem + vancomycin documented (exact doses unavailable)	Persistent deep infection/FRI. Repeated *B. fragilis* recovery increased its clinical plausibility; other isolates were interpreted with specimen and operative context.
20 May–1 June 2026 (after transfer)	Prior culture results used to guide treatment	Vancomycin 0.5 g IV q12h (20–26 May); norvancomycin 0.8 g IV q12h (26–28 May); levofloxacin 0.5 g IV q24h (27 May–1 June); host and wound optimization	Therapeutic rather than prophylactic antimicrobial treatment was ongoing while definitive source-control planning proceeded.
4 June 2026: repeat debridement	Subsequent reports included *Bacillus cereus* and *Bacillus subtilis*	Removal of previous cement; repeat radical debridement and deep sampling; temporary vancomycin-loaded PMMA; VAC bridge	Spore-forming organisms may represent infection, colonization, or contamination; specimen source and repeated recovery are important.
22 June–13 July 2026: peri-reconstruction period	Later reports included *Paenibacillus* spp., *Staphylococcus haemolyticus*, and *Staphylococcus warneri*	Levofloxacin 0.5 g IV q24h (22 June–13 July); linezolid 600 mg IV q12h (23–30 June); temporary PMMA removed and repeat debridement performed on 25 June before flap coverage	Dynamic polymicrobial profile under repeated surgery and antibiotic pressure; not every new isolate was considered a discrete new infection.
Follow-up	No single organism was assumed to explain every fluctuation in inflammatory markers	Serial wound review and inflammatory-marker monitoring (CRP/ESR/IL-6)	Clinical wound status and inflammatory trends were interpreted together during follow-up.

**Table 2:** Clinical timeline.

Date/Period	Clinical Event	Key Findings/Treatment
Mid-March 2026 (approx.)	Industrial open injury	Angle-grinder laceration of the right lower leg causing a localized incomplete open tibial fracture; subsequent imaging showed preserved tibial alignment and longitudinal continuity without major displacement or a large segmental osseous defect; initial simple debridement and primary closure.
3 April 2026	First major reoperation	Debridement, cement filling/coverage of the local wound dead space, and VAC.
10 April 2026	Repeat debridement	Debridement, cement-spacer placement, and VAC.
17 April 2026	Local flap reconstruction	Debridement and soleus muscle-flap transfer; subsequent flap necrosis/infection.
11–18 May 2026	Tertiary-center treatment	Further tibial/soft-tissue debridement and antibiotic cement; meropenem + vancomycin documented. Serial cultures: *C. tuberculostearicum* (12 May), *B. fragilis* (13 May), and *B. fragilis* + *S. epidermidis* (16 May); CRP increased to 64.1 mg/L on 18 May.
20 May 2026	Transfer to orthoplastic unit	~8 cm × 6 cm defect with cement coverage, edema, erythema, and drainage; severe anemia, elevated inflammatory markers, and suboptimal glycemic control.
4 June 2026	Stage 1 source control	Removal of prior cement, repeat radical debridement, deep cultures, temporary vancomycin-loaded PMMA, and VAC bridge.
18–22 June 2026	Interval optimization	Hemoglobin improved to ~115 g/L; CRP ~6.6–7.4 mg/L; host, wound, skeletal alignment, and reconstructive readiness reassessed.
25 June 2026	Definitive vascularized soft-tissue coverage	Temporary PMMA removed; repeat debridement; ~16 cm × 7 cm defect reconstructed using chimeric ALT skin paddle + vascularized vastus lateralis muscle; anterior tibial recipient vessels; sensory nerve coaptation.
3 July 2026	Early laboratory follow-up	ESR ~15 mm/h; CRP ~7.3 mg/L.
11 July 2026	Transient inflammatory rebound	ESR 120 mm/h and CRP 44.3 mg/L; no documented flap compromise, recurrent wound breakdown, or drainage.
13 July 2026	Hospital discharge	Clinically stable with a viable flap; discharged from our center and transferred to a lower-level hospital for continued systemic antimicrobial therapy.
10 August 2026	Short-term outpatient follow-up	Flap viable with stable coverage; CRP 6.9 mg/L, ESR 44 mm/h, Hgb 112 g/L; radiographs showed preserved tibial alignment and longitudinal continuity with a stable appearance of the localized incomplete fracture.
30 August 2026	Latest short-term outpatient follow-up (~9.5 weeks after reconstruction)	Chimeric ALT flap fully viable with stable soft-tissue coverage; CRP 2.0 mg/L and IL-6 3.8 pg/mL within institutional reference ranges; ESR 22 mm/h, close to the reference limit; marked overall inflammatory improvement.
Ongoing	Planned surveillance	Continued clinical, laboratory, and radiographic follow-up for recurrent infection, fracture healing, weight-bearing recovery, sensory recovery, and limb function.

## Data Availability

The clinical information supporting this case report is contained within the article. Additional de-identified information may be available from the corresponding author upon reasonable request, subject to institutional approval and patient privacy requirements.

## Supplementary Materials

The following supporting information can be downloaded at: https://www.mdpi.com/article/10.3390/jcm15207804/s1. Completed CARE checklist for this case report.

## Abbreviations

The following abbreviations are used in this manuscript:

ALT	anterolateral thigh
CTA	computed tomography angiography
CoNS	coagulase-negative staphylococci
CRP	C-reactive protein
ESR	erythrocyte sedimentation rate
FRI	fracture-related infection
LCFA	lateral circumflex femoral artery
NPWT	negative-pressure wound therapy
PMMA	polymethylmethacrylate
VAC	vacuum-assisted closure
